# DNA polymerase κ suppresses inflammation and inflammation-induced mutagenesis and carcinogenic potential in the colon of mice

**DOI:** 10.1186/s41021-023-00272-7

**Published:** 2023-04-22

**Authors:** Atsushi Hakura, Hajime Sui, Yuki Seki, Jiro Sonoda, Yusaku Yoshida, Hisayoshi Takagi, Shigeo Yokose, Tomonari Matsuda, Shoji Asakura, Takehiko Nohmi

**Affiliations:** 1 Global Drug Safety, Eisai Co., Ltd., 5-1-3 Tokodai, Tsukuba-Shi, Ibaraki, 300-2635 Japan; 2 Division of Safety Testing, Food and Drug Safety Center, Hatano Research Institute, Hadano, Kanagawa 257-0025 Japan; 3 Present Address: Operations Department, Global Safety HQS, Eisai Co., Ltd., 4-6-10 Koishikawa, Bunkyo-Ku, Tokyo, 112-8088 Japan; 4 Biotechnical Center, Japan SLC, Inc., 3-5-1 Aoihigashi, Naka-Ku, Hamamatsu-Shi, Shizuoka, 433-8114 Japan; 5 Research Center for Environmental Quality Management, Kyoto University, Otsu, Shiga 520-0811 Japan; 6 Division of Pathology, National Institute of Health Sciences, 3-25-26 Tonomachi, Kawasaki-Ku, Kawasaki-Shi, Kanagawa, 210-9501 Japan

**Keywords:** Chronic inflammation, DNA polymerase κ, Translesion DNA synthesis, Colitis, Dysplasia, In vivo mutation, DNA damage

## Abstract

**Background:**

Chronic inflammation induces DNA damage and promotes cell proliferation, thereby increasing the risk of cancer. DNA polymerase κ (Pol κ), involved in translesion DNA synthesis, counteracts mutagenesis induced by inflammation in the colon of mice. In the present study, we examined whether Pol κ suppressed inflammation-induced colon tumorigenesis by treating inactivated *Polk* knock-in (*Polk*^−/−^) mice with dextran sulfate sodium (DSS), an inducer of colon inflammation.

**Results:**

Male and female *Polk*^−/−^ and *Polk*^+/+^ mice were administered 2% DSS in drinking water for six consecutive days, succeeded via a recovery period of 16 days, followed by 2% DSS for another two days. DSS treatment strongly induced colitis, and the severity of colitis was higher in *Polk*^−/−^ mice than in *Polk*^+/+^ mice. The mice were sacrificed after 19 weeks from the initiation of the first DSS treatment and subjected to pathological examination and mutation analysis. DSS treatment induced colonic dysplasia, and the multiplicity of dysplasia was higher in *Polk*^−/−^ mice than in *Polk*^+/+^mice. Some of the dysplasias in *Polk*^−/−^ mice exhibited β-catenin-stained nucleus and/or cytoplasm. Mutation frequencies in the *gpt* reporter gene were increased by DSS treatment in *Polk*^−/−^ mice, and were higher than those in *Polk*^+/+^ mice.

**Conclusions:**

Pol κ suppresses inflammation and inflammation-induced dysplasia as well as inflammation-induced mutagenesis. The possible mechanisms by which Pol κ suppresses colitis- and colitis-induced dysplasia are discussed.

**Supplementary Information:**

The online version contains supplementary material available at 10.1186/s41021-023-00272-7.

## Introduction

Chronic inflammation is a known risk factor for human cancer. Crohn’s disease and ulcerative colitis are chronic inflammatory bowel diseases strongly associated with a high risk of intestinal cancer [1, 2]. The inhibition of inflammation in patients using anti-inflammatory drugs substantially reduces cancer risk. The colonization of *Helicobacter pylori* in the epithelium of the stomach is another example of chronic inflammation-induced human cancer [3]. During inflammation, reactive oxygen and nitrogen species, and lipid peroxidation products are generated, which induce a variety of DNA adducts [4]. Inflammation also generates various inflammatory mediators, such as cytokines, chemokines, and matrix-degrading enzymes, which may promote cell proliferation and carcinogenesis [5]. Thus, DNA damage, mutagenesis, and cell proliferation play important roles in inflammation-induced carcinogenesis.

When genomic DNA is damaged, the lesions are initially repaired by several cellular DNA repair mechanisms, such as nucleotide excision repair and base excision repair [6]. However, all DNA damage is not repaired before DNA replication. Therefore, replicative DNA polymerases (Pols), such as Pol α, Pol δ, or Pol ε, encounter DNA damage and halt DNA synthesis, which may lead to chromosome aberrations and cell death [7]. In this case, cells adopt a strategy called translesion DNA synthesis (TLS) [8–10]. In this process, specialized Pols for TLS incorporate dNMPs opposite DNA damage, extend the primer DNA, and hand over the primer to the replicative Pols to complete whole chromosome replication. Unlike replicative Pols, which synthesize long stretches of chromosome DNA with high fidelity, specialized Pols synthesize short DNA that often contains DNA damage with low fidelity [11]. Typical examples of specialized Pols are Pol κ, Pol η, Pol ι, and Rev1, which belong to the Y-family, and Pol ξ, a B-family enzyme [12, 13]. Although TLS rescues halted chromosome replication, it does not remove DNA damage, but only helps complete chromosome replication. Thus, TLS is regarded as a mechanism of DNA damage tolerance rather than DNA repair [14].

Among the specialized Pols, Pol κ is unique in that its homologs are present in three domains of life, i.e., Eukarya, Archaea, and Bacteria, whereas others are present either in Eukarya or in Bacteria/Archaea [10, 15]. To elucidate the in vivo protective roles against genotoxic insults, we generated knock-in mice whose catalytic activity of Pol κ was inactivated [16] and crossed them with *gpt* delta mice [17], which have a reporter gene for mutations (hereafter, we refer to the inactivated *Polk*^−/−^ knock-in *gpt* delta mice simply *Polk*^−/−^ mice). We then examined the sensitivity of *Polk*^−/−^ mice to mutagenicity and carcinogenicity of benzo[*a*]pyrene (BP) via intragenic gavage, followed by treatment with dextran sulfate sodium (DSS), and compared the mutant frequencies (MFs) and tumor formation in the colon with those of *gpt* delta mice (hereafter, we refer to the mice simply *Polk*^+/+^ mice). BP was chosen as a genotoxic insult because Pol κ efficiently bypasses *N*^2^-2’-deoxyguanosine adducts by active metabolites of BP in an error-free manner in vitro [18–21]. DSS accelerates chemically-induced carcinogenesis by inducing inflammation in the mouse colon [22, 23]. However, *Polk*^−/−^ mice did not exhibit any substantially higher sensitivity to the mutagenicity of BP and carcinogenicity of BP + DSS in the colon compared to *Polk*^+/+^ mice [24, 25]. Instead, *Polk*^−/−^ mice exhibited much higher sensitivity to the mutagenicity of DSS than *Polk*^+/+^ mice. These results were surprising because DSS was known to be non-mutagenic both in vitro and in vivo [26, 27]. In fact, DSS did not induce mutations in *Polk*^+/+^ mice in a previous study [24]. Since DSS strongly induces inflammation in the colon, we suggest that Pol κ bypasses inflammation-induced DNA damage(s) in an error-free manner, thereby suppressing inflammation-induced mutagenesis. This result also raised the interesting possibility that *Polk*^−/−^ mice might be sensitive to inflammation-induced carcinogenesis.

In this study, we investigated the possibility by oral treatment of *Polk*^−/−^ and *Polk*^+/+^ mice with DSS alone, and compared their sensitivity to DSS-induced inflammation, dysplasia, and mutagenesis in the colon. These results suggest that Pol κ suppresses inflammation, inflammation-induced dysplasia, and mutagenesis in the colon of mice. Possible mechanisms behind the suppression of inflammation and inflammation-induced genome instability by Pol κ are discussed.

## Materials and methods

### Chemicals

DSS (CAS No. 9011–18-1, molecular weight:36,000–50,000) was obtained from MP Biochemicals LLC (Aurora, OH, USA).

### Animals

**Fig. 1:**
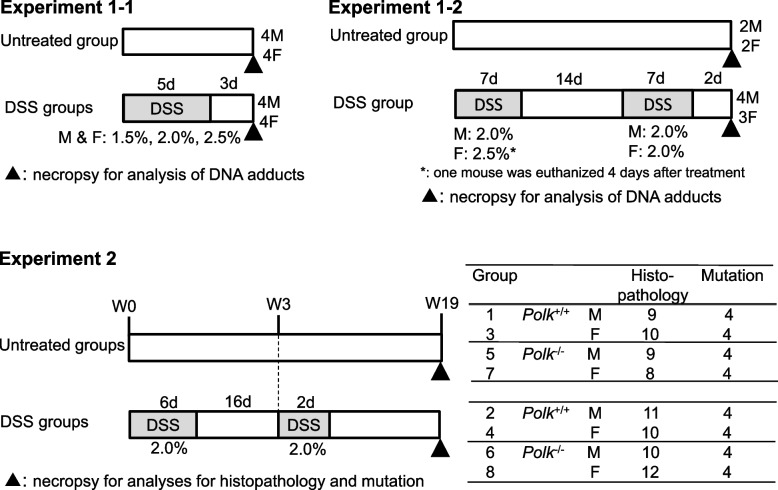
Experimental designs. Experiment 1–1 was for a dose-finder for Experiment 1–2 and sampling of the colon mucosa, and three concentrations (1.5%, 2.0%, and 2.5%) of DSS were tested for male and female C57BL/6 J mice. Four males and four females from the untreated and DSS groups were used to analyzing the DNA adducts. Based on Experiment 1–1, the concentrations of 2.0% and 2.5% DSS were initially selected for male and female C57BL/6 J mice in Experiment 2, respectively. As one female mouse was euthanized due to poor health conditions after treatment with DSS, the concentration of DSS was reduced from 2.5% to 2.0%. Colon mucosa was collected from two males and two females from the untreated group, and four males and three females from the DSS group to analyze DNA adducts. The treatment regimen of Experiment 2 was determined based on the data from Experiment 1–2, and animal conditions such as clinical signs, stool, and body weight so that mortality might be minimal. Male and female *Polk*^+/+^ and *Polk*^−/−^ mice were administered 2.0% DSS for six consecutive days and two consecutive days, separated by a 16-day DSS treatment-free interval period. At week 19 (defining the first week of the experiment as week 0), surviving mice were necropsied to collect colon samples for histopathology and mutation assays, as indicated in the text table

*Polk*^−/−^ mice were established as described [16]. Briefly, *Polk* mutant ES cells with a DNA sequence that directed amino acid substitutions of D197A-E198A were injected into BALB/cA blastocytes to produce chimeric mice. Chimeric mice were bred with C57BL/6N females to generate their offspring. After homogenization of the *Polk* mutant allele, the knock-in mice were bred with C57BL/6 J-Tg *gpt* delta mice harboring the *gpt* gene for mutation [17]. *Polk*^+/+^ and *Polk*^−/−^ mice were bred at Japan SLC Inc. (Shizuoka, Japan). C57BL/6 J mice used for Experiment 1–1 and 1–2 (Fig. 1) were purchased from SLC, Inc. (Shizuoka, Japan).

The mice were transported to the test site (Eisai Co., Ltd., Ibaraki, Japan). All mice were housed in plastic cages (one mouse per case) and fed a basal diet (Oriental MF, Oriental Yeast Co., Ltd., Tokyo, Japan) and tap water (Experiment 1) (Fig. 1) or purified water (Experiment 2) (Fig. 1) ad libitum under controlled conditions of temperature (23 ± 3 °C), humidity (55 ± 15%), and light (12-h light/12-h dark cycle). Animals were quarantined for one week or more, and they were assigned by stratified randomization to the two groups according to their body weights. Mice from the DSS- and untreated groups were assigned to the heavy and light groups, respectively, to minimize the mortality of light mice, given that they are likely to be more sensitive to DSS-induced colitis than heavy mice.

### Experimental procedures

The present study comprised two experiments (Experiments 1 and 2) (Fig. 1). Experiment 1 was conducted to determine an appropriate concentration of DSS in Experiment 2 using C57BL/6 mice, which was identical to *Polk*^+/+^ and *Polk*^−/−^ mice, and to analyze DSS-induced DNA adducts in the colon mucosa obtained at necropsy. Experiment 2 examined the role of Pol κ in tumorigenesis and mutagenesis induced by DSS using *Polk*^+/+^ and *Polk*^−/−^ mice.

#### Experiment 1

Experiment 1 was subdivided into two experiments (Experiments 1–1 and 1–2) (Fig. 1). In Experiment 1–1, four male and four female mice (9-weeks old) per group were administered 1.5%, 2.0%, or 2.5% DSS in drinking water for five consecutive days, and necropsied three days after the last DSS treatment to prepare colonic mucosa samples for analysis of DNA adducts. Four male and four female mice were used as untreated controls.

The purpose of Experiment 1–2 was to determine an appropriate concentration of DSS for Experiment 2, where two cycles of DSS treatment for one week separated by a two-week DSS treatment-free interval period were initially scheduled. Based on the results from Experiment 1–1, the concentration of DSS was initially set at 2.0% for four males and 2.5% for four females; however, the concentration for females was decreased to 2.0% for the second cycle because one female was euthanized due to poor health conditions caused by colitis following four days after the first cycle of DSS treatment. Two days after the last treatment in the second cycle, the mice were necropsied for sampling. Two male and two female mice were used as untreated controls for the analysis.

In Experiments 1–1 and 1–2, after the mice were euthanized by exsanguination from the abdominal aorta or vein under isoflurane anesthesia, the large intestine was immediately excised and flushed with saline. The colonic mucosa was scraped using a spatula. After being snap frozen, samples were stored in a deep freezer until DNA extraction for the analysis of DNA adducts.

#### Experiment 2

The treatment regimen of Experiment 2 was determined based on the data from Experiment 1–2, and animal conditions, i.e., stool and body weight. Actually, 16 male *Polk*^+/+^, female *Polk*^+/+^, male *Polk*^−/−^, or female *Polk*^−/−^ mice (7 to 9-weeks old) per group were administered 2.0% DSS in drinking water for six consecutive days and two consecutive days separated by a 16-day DSS treatment-free interval period. As a reference, purified water was given to 12–14 male *Polk*^+/+^, female *Polk*^+/+^, male *Polk*^−/−^, or female *Polk*^−/−^ mice per group. At week 19 (defining the first week of the experiment as week 0), mice were euthanized by exsanguination from the abdominal aorta or vein under isoflurane anesthesia to collect colon samples for histopathology and mutation assays.

### Tissue collection and histopathology

Since five mice treated with DSS were found dead or sacrificed due to poor physical conditions, 11 male *Polk*^+/+^, 10 female *Polk*^+/+^, 10 male *Polk*^−/−^ or 12 female *Polk*^−/−^ mice for DSS treatment, and 9 male *Polk*^+/+^, 10 female *Polk*^+/+^, 9 male *Polk*^−/−^ or 8 female *Polk*^−/−^ mice as vehicle controls were examined histopathologically, as described previously [23, 28]. At necropsy, the large intestine was immediately excised, flushed with saline, infused with 10% neutral buffered formalin, cut open longitudinally, and grossly examined. Thereafter, the tissues were stored in 10% neutral buffered formalin, cut into four equal lengths from the proximal to the distal ends, processed, and embedded in paraffin.

Longitudinal sections of the colon were stained with hematoxylin and eosin (H&E) for histopathological examination, and the number of colonic dysplasia (consisting of multiple dysplastic foci), adenomas, and adenocarcinomas were counted. These dysplastic and neoplastic lesions were diagnosed according to previously described criteria [23, 28], which were originally reported by Pascal [29], Riddell et al. [30] and Ward [31].

The study design was approved by the Institutional Animal Care and Use Committee, and the experiments were performed according to Eisai animal experimentation regulations.

### Immunohistochemistry

Paraffin-embedded colon sections with dysplastic lesions in DSS-treated mice were examined using immunohistochemical staining for β-catenin. Rabbit monoclonal anti-β-catenin antibody (clone E247, Abcam) was used at a concentration of 1/1000. After autoclave antigen retrieval of 4 µm sections, DAKO EnVision + System-HRP Labelled Polymer Anti-Rabbit was used to examine their expression and localization. The sections were counterstained with Mayer’s hematoxylin solution for microscopic examination.

### Tissue collection for mutation assay

Four mice from each of the eight groups (male *Polk*^+/+^, female *Polk*^+/+^, male *Polk*^−/−^ and female *Polk*^−/−^ mice treated with or without DSS) were used in the mutation assay. At necropsy, the large intestine was immediately excised, flushed with saline, and the mucosa was scraped from the distal colon (half of the anus side) using a spatula. After being snap frozen, samples were stored in a deep freezer until DNA extraction for mutation assay.

### Mutation assay

MFs of the *gpt* reporter gene were determined using 6-thioguanine (6-TG) selection according to a previous report [17]. Initially, isolated *gpt* mutants were confirmed by streaking them on agar plates containing 6-TG. Then, the "confirmed mutants" were subjected to DNA sequencing. When mutations were identified, they were classified as "corrected mutants". The MFs were calculated by dividing the number of "corrected mutants" by the number of rescued phages (Table 3). Identical mutations obtained from the same mouse were regarded as single mutations because of clonal expansion.

### DNA adduct analyses

DNA adducts in the colonic mucosa of DSS-treated or non-treated mice were quantified by LC/MS/MS, as described previously, with minor modifications [32]. Lipid peroxidation-derived DNA adducts, 1,*N*^6^-etheno-2′-deoxyadenosine (εdA), 8-hydroxy-1, *N*^2^-propano-2′-deoxyguanosine (8-OH-AdG), heptanone-etheno-2′-deoxycytidine (HεdC), and an oxidative DNA adduct 8-oxo-7,8-dihydro-2’-deoxyguanosine (8-oxo-dG) were analyzed.

DNA was extracted from the colonic mucosa of the mice using the QIAGEN Blood and Tissue Kit, as indicated in the kit protocol. The DNA sample (~ 10 μg) was digested to deoxynucleosides by micrococcal nuclease, spleen phosphodiesterase, and alkaline phosphatase, as previously described [32]. Digested DNA was dissolved in 25 μL of 30% dimethyl sulfoxide, and a 2 μL aliquot was injected and separated by the CORTECS™ UPLC® C18 1.6 µm 2.1 × 100 mm column (Waters) heated to 40 °C, eluted in a linear gradient of 1%–10% methanol in 10 mM ammonium acetate buffer from 0 to 4 min, 10% to 80% from 4 to 8 min, at a flow rate of 0.2 mL/min. Multiple reaction monitoring was performed using a Waters Xevo TQS quadrupole tandem mass spectrometer with electrospray ionization. The MS parameters were as follows in electrospray positive mode: cone, 35 V; source temperature, 150 °C; desolvation temperature, 650 °C; cone gas flow, 150 L/h; desolvation gas flow, 1000 L/h; collision gas flow, 0.15 mL/min; and nebulizer gas flow, 7 bar. The target substance was measured using multiple reaction monitoring (MRM). The MRM settings for each adduct are as follows (notation: parental ion (m/z) → daughter ion (m/z); collision energy (V)): 8-oxo-dG (284.1 → 168.1; 15), [^15^N_5_]8-oxo-dG (289.1 → 173.1; 15), εdA (276.1 → 160.1; 15), [^15^N_5_]εdA (281.1 → 165.1; 15), 8-OH-AdG (324.1 → 208.1; 10), [^15^N_5_]8-OH-AdG (329.1 → 213.1; 10), HεdC (364.2 → 248.2; 10), [^15^N_5_] HεdC (367.2 → 251.2; 10).

### Statistical analyses

The total number of days when mice showed blood stool, soft stool, or decreased body weight was compared using a two-sided Welch’s *t*-test for paired samples. The incidence and multiplicities of dysplasia were compared using Fisher’s exact probability test and Welch’s *t*-test for paired samples (two-sided both). MFs were compared using a two-sided Dunnett test for paired samples.

## Results

### Colitis activity indices

We examined the appearance of blood or soft stools and decrease in body weight during the in-life phase, and the length of the large intestine at necropsy as signs of inflammation-related findings (Table 1, Supplementary Table 1). In this study, the number of days mice showed bloody stool, soft stool, or decreased body weight, was used as a colitis activity index for inflammation induced in the colon. Blood or soft stools, or decreased body weights were observed in both male and female mice, and both *Polk*^−/−^ and *Polk*^+/+^mice treated with DSS. In many mice, blood or soft stools appeared particularly for 4 days after the first DSS treatment, 2 days from the last day of the second DSS treatment, or 3 weeks from one week after the second DSS treatment. The body weight decreased in approximately half of the mice in each group (both males and females and both *Polk*^−/−^ and *Polk*^+/+^mice) treated with DSS, particularly for 4 or 5 days from 2 days after the first DSS treatment. When we compared the colitis activity indices between *Polk*^−/−^ and *Polk*^+/+^ mice (Table 1), they were all higher in *Polk*^−/−^ mice than in *Polk*^+/+^mice, and the difference in soft stools in females was statistically significant. This finding indicates that *Polk*^−/−^ mice may be more sensitive to DSS-induced inflammation than *Polk*^+/+^mice and that Pol κ may be closely associated with inflammation. In contrast, DSS treatment did not affect the length of the large intestine, and there were no differences in the length between *Polk*^−/−^ and *Polk*^+/+^mice or between males and females.

**Table 1:**
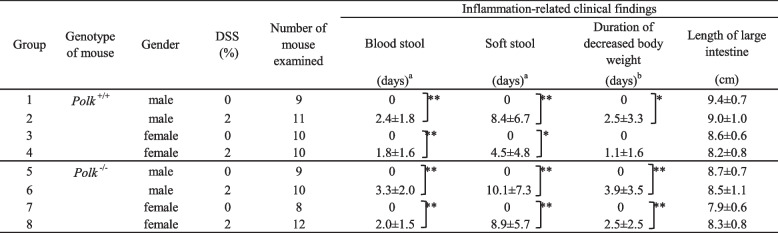
Inflammation-related findings in untreated or DSS-treated *Polk*^+/+^ and *Polk*^−/−^ mice

^*^Significantly different at *p* < 0.05

^**^Significantly different at *p* < 0.01

^a^Number of days when blood or soft stools were observed

^b^Number of days when body weights decreased by 10% or more as compared to the maximum body weight during the most recent dosing period

### Histopathology

**Fig. 2:**
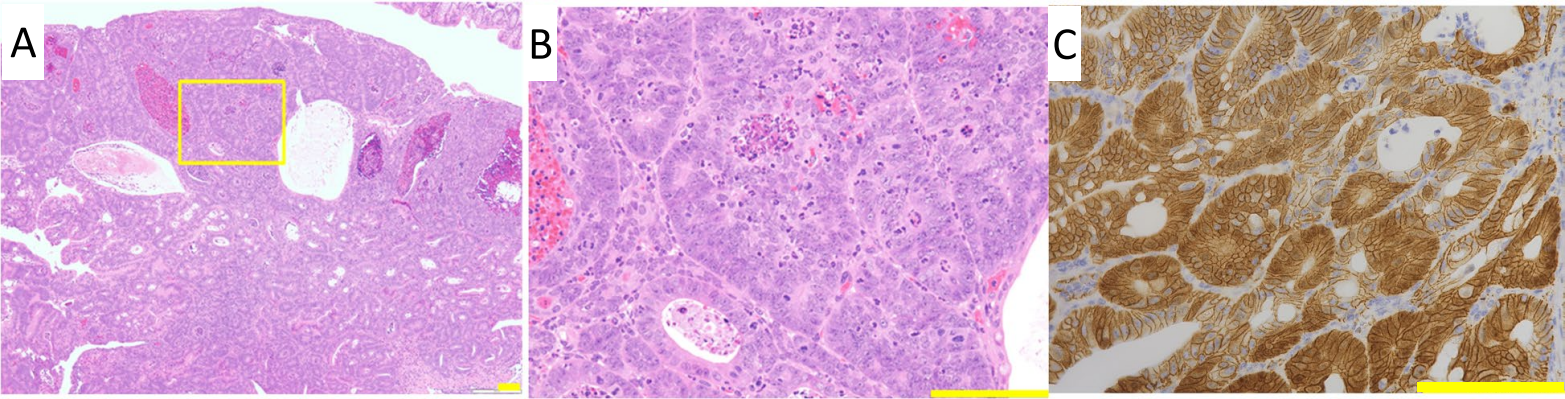
Histopathology results of H&E staining and β-catenin-immunostaining of adenocarcinoma in the colon induced by DSS. **A** and **B** show adenocarcinoma (Mouse ID; 06M07) induced in male *Polk*^−/−^ mouse after DSS treatment. β-Catenin is accumulated in the nucleus or cytoplasm of the adenocarcinoma (Fig. 2**C**). **B** is enlarged magnification of the area surrounding the yellow frame in Fig. 2**A**. bar: 100 µm

**Fig. 3:**
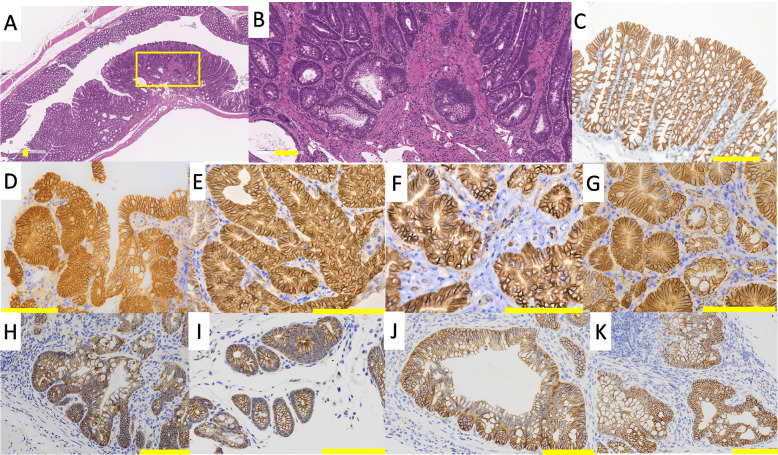
Histopathology results of H&E staining and β-catenin-immunostaining of dysplasias in the colon induced by DSS. **A** and **B** show dysplasia stained with H & E (Mouse ID; 02M11) induced in a male *Polk*^−/−^ mouse after DSS treatment. **C** to **K** show colorectal epithelium or dysplasias stained with β-catenin. The β-catenin is exclusively located on the cell membrane in normal colorectal epithelium (Fig. 3**C**). Some dysplasias induced in female *Polk*^−/−^ mice, where β-catenin is accumulated in the nucleus or cytoplasm, are shown in Figs. 3**D** (Mouse ID; 08F06), 3**E** (Mouse ID; 08F07), 3**F** (Mouse ID; 08F10), and 3**G** (Mouse ID; 08F10). All dysplasias examined in male *Polk*^+/+^ and *Polk*^−/−^ mice show localization of β-catenin limited to the cell membrane, and representative images of are shown in Figs. 3**H** (Mouse ID; 02M08) and 3**I** (Mouse ID; 02M09) for *Polk*^+/+^mice, and in Figs. 3**J** (Mouse ID; 06M11) and 3 **K** (Mouse ID; 06M12) for *Polk*^−/−^mice. Figure 3**B** is enlarged magnification of the area surrounding the yellow frame in Fig. 3**A**. bar: 100 µm

Inflammatory cell infiltration was observed in both *Polk*^−/−^ and *Polk*^+/+^ mice 15 weeks after the initial DSS treatment (data not shown), indicating that colitis was induced by DSS treatment. Dysplastic foci were characterized by irregular branching with cellular and nuclear pleomorphisms (Figs. 3A and B). One adenocarcinoma was a polypoid mass composed of tubular or papillary proliferations that protruded into the intestinal lumen and consisted of variably sized distorted glands, which branched irregularly and were lined by stratified epithelial cells with cellular and nuclear pleomorphism (Figs. 2A and B). These histopathological characteristics are consistent with those found in various DSS-induced colon cancer models. There were no differences in the histopathological characteristics of the colon mucosa of *Polk*^−/−^ and *Polk*^+/+^ mice treated with vehicle.

### Induction of dysplastic and neoplastic lesions in the colon by DSS

Under the treatment conditions employed in the present study, 30 dysplasias (dysplastic foci) and one adenocarcinoma were observed in the colon of 43 *Polk*^+/+^ and *Polk*^−/−^ mice treated with DSS (Supplementary Table 1). There were no colonic dysplasia or neoplastic lesions in 36 *Polk*^+/+^ and *Polk*^−/−^ mice treated with vehicle.

*Polk*^−/−^ mice exhibited a higher multiplicity of colonic dysplasia and neoplastic lesions than *Polk*^+/+^ mice, and the difference in the multiplicity of dysplasia was statistically significant in females (Table 2). These findings suggest that Pol κ suppresses DSS-induced tumorigenesis. In both *Polk*^+/+^ and *Polk*^−/−^ mice treated with DSS, males exhibited higher multiplicity than females in dysplasia. The higher sensitivity of males than females is consistent with the higher sensitivity of males for colitis activity indices (Table 1).

**Table 2:**
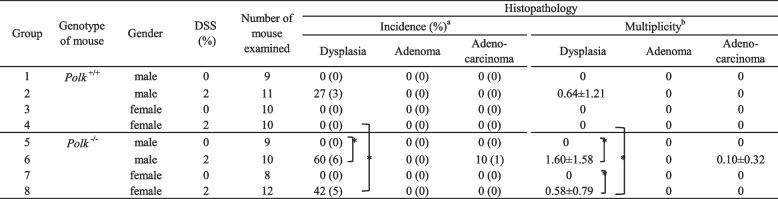
Colonic dysplasias and neoplasias in untreated or DSS-treated *Polk*^+/+^ and *Polk*^−/−^ mice

^*^Significantly different at *p* < 0.05

^a^Figures in parentheses indicate the number of dysplasia/tumor-bearing mice

^b^Number of dysplasia/tumors per mouse, mean ± standard deviation

### Immunohistochemistry of β-catenin in dysplastic foci

The translocation and accumulation of β-catenin from the cell membrane to the nucleus are associated with colon tumorigenesis in rodents and humans. Such abnormal β-catenin-accumulated dysplastic foci are considered advanced dysplasia or putative preneoplastic lesions of the colon in chemically-induced colon cancer models in combination with or without DSS [33]. For this reason, we conducted a β-catenin-immunohistochemical analysis of the majority of the dysplasia cases from mice exposed to DSS, which were available in serial sections of H &E specimens, i.e., five dysplasias from male *Polk*^+/+^ mice, 12 from male *Polk*^−/−^ mice, and seven from female *Polk*^−/−^ mice. β-catenin was exclusively localized on the cell membrane in the normal colonic mucosal epithelial cells of vehicle-treated mice (Fig. 3C). Likewise, all the dysplasias examined in male *Polk*^+/+^ and *Polk*^−/−^ mice showed localization of β-catenin in cell membrane (Figs. 3H to K). However, β-catenin was present in the nucleus and/or cytoplasm in four out of seven dysplasias in female *Polk*^−/−^ mice (Figs. 3D to G) as well as one adenocarcinoma in a male *Polk*^−/−^ mouse (Fig. 2C). Two out of seven dysplasias did not show clear translocation of β-catenin and the remaining one showed localization of β-catenin in cell membrane. These findings suggest that at least some dysplastic foci were possible neoplastic lesions.

### Induction of mutations in the colon of *Polk*^−/−^ mice by DSS

MFs of the *gpt* reporter gene were determined in the colons of female *Polk*^+/+^ and *Polk*^−/−^ mice treated with DSS or vehicle (Table 3, Supplementary Fig. 1). DSS enhanced MFs almost twice in *Polk*^−/−^ mice (10.3 ± 6.3 × 10^–6^ versus 5.2 ± 3.0 × 10^–6^), while it did not increase MFs in *Polk*^+/+^ mice (4.0 ± 1.5 × 10^–6^ versus 4.4 ± 2.0 × 10^–6^). Thus, DSS-induced MF was higher in *Polk*^−/−^ mice than in *Polk*^+/+^ mice (10.3 ± 6.3 × 10^–6^ versus 4.0 ± 1.5 × 10^–6^, although there was no statistical significance. To analyze the types of mutations that were increased in DSS-treated *Polk*^−/−^ mice, mutation spectra were compared between DSS-treated *Polk*^+/+^ and *Polk*^−/−^ mice. Specific MFs of the A:T to T:A transversions and one-base deletions were five times or higher in *Polk*^−/−^ than in *Polk*^+/+^ mice (Table 4). The specific MFs of A:T to G:C and G:C to T:A were more than three times higher in *Polk*^−/−^ than in *Polk*^+/+^ mice. In contrast, there were no substantial differences in the mutation spectra between the water-treated *Polk*^+/+^ and *Polk*^−/−^ mice.

**Table 3:** *gpt* MF in the distal colon of untreated or DSS-treated *Polk*^+/+^ and *Polk*^−/−^ female mice

**Group**	**Genotype**		**Total population**	**Confirmed mutants**^**a**^	**Mutant Frequency (× 10**^**–6**^**)**	**Mutant Frequency (× 10**^**–6**^**) (average ± S.D.)**	**Sequenced mutants**	**Corrected mutants**^**a**^	**M F**^**a**^** (× 10**^**–6**^**)**	**M F (× 10**^**–6**^**) (average ± S.D.)**
3	*Polk*^+/+^	0	990,000	4	4.04	5.2 ± 1.8	4	2	2.0	4.4 ± 2.0
		0	1,344,000	5	3.72		5	5	3.7	
		0	786,000	4	5.09		4	4	5.1	
		0	900,000	7	7.78		7	6	6.7	
4	*Polk*^+/+^	2	1,674,000	5	2.99	5.3 ± 3.0	5	5	3.0	4.0 ± 1.5
		2	816,000	2	2.45		2	2	2.5	
		2	960,000	8	8.33		7	5	5.2	
		2	966,000	7	7.25		7	5	5.2	
7	*Polk*^−/−^	0	2,940,000	27	9.18	6.8 ± 4.1	25	18	6.1	5.2 ± 3.0
		0	792,000	3	3.79		3	2	2.5	
		0	1,014,000	3	2.96		3	3	3.0	
		0	1,668,000	19	11.39		19	15	9.0	
8	*Polk*^−/−^	2	1,440,000	35	24.31	17.8 ± 12.3	34	15	10.4	10.3 ± 6.3
		2	1,566,000	16	10.22		15	15	9.6	
		2	984,000	31	31.50		31	18	18.3	
		2	3,762,000	19	5.05		19	11	2.9	

^a^Genomic DNA was extracted from distal portion of the large intestine of *Polκ*^+/+^ and *Polκ*^−/−^ female mice and subjected to *gpt* assays. Initially isolated *gpt* mutants were confirmed by streaking them on agar plates containing 6-TG. Then, the "confirmed mutants" were subjected to DNA sequencing. When the mutations were identified, they were classified as "corrected mutants". "MF" was calculated by dividing the number of "corrected mutants" by the number of "total population"

**Table 4:** Specific *gpt* MF in distal colon of untreated- or DSS-treated *Polk*^+/+^ and *Polk*^−/−^ mice

	**Water**							**DSS**						
	**Polk**^**+/+**^			**Polk**^**−/−**^				**Polk**^**+/+**^			**Polk**^**−/−**^			
	**No. of**	**%**	**SMF**	**No. of**	**%**	**SMF**	**Polk**^**−/−**^** / Polk**^**+/+**^** (Fold)**	**No. of**	**%**	**SMF**	**No. of**	**%**	**SMF**	**Polk**^**−/−**^** / Polk**^**+/+**^** (Fold)**
	**mutant**		**(× 10**^**–6**^**)**	**mutant**		**(× 10**^**–6**^**)**		**mutant**		**(× 10**^**–6**^**)**	**mutant**		**(× 10**^**–6**^**)**	
Base substitution														
Transition														
G:C to A:T	6	35	1.6	14	37	1.9	1.2	6	33	1.3	18	29	2.9	2.2
A:T to G:C	2	12	0.5	2	5	0.3	0.6	1	6	0.2	5	8	0.8	4.0
Transversion														
G:C to T:A	6	35	1.6	7	18	1.0	0.6	4	22	0.9	17	27	2.8	3.1
G:C to C:G	0	0	0.0	3	8	0.4		2	11	0.4	5	8	0.8	2.0
A:T to T:A	0	0	0.0	6	16	0.8		1	6	0.2	7	11	1.1	5.5
A:T to C:G	2	12	0.5	2	5	0.3	0.6	1	6	0.2	3	5	0.5	2.5
Deletion														
1 base pair	1	6	0.3	2	5	0.3	1.0	1	6	0.2	6	10	1.0	5.0
> 2 base pairs	0	0	0.0	1	3	0.1		1	6	0.2	1	2	0.2	1.0
Insertion	0	0	0.0	1	3	0.1		1	6	0.2	1	2	0.2	1.0
Total	17	100	4.4	38	100	5.2	1.2	18	100	4.0	63	100	10.3	2.6

^a^SMF stands for specific MF, which was calculated by multiplying the MF by the ratio of the number of mutations in each class among the total number of *gpt* mutations

### DSS-induced DNA adducts in the colon of mice

We measured the amount of four different types of DNA adducts in the colon mucosa of male and female C57BL/6 mice exposed to DSS (Fig. 1, Table 5). The amount of εdA increased slightly compared to the control level when the female mice were exposed to two cycles of 2.5 or 2.0% DSS for 7 days (1.4 adducts per 10^8^ bases versus 0.8 adducts per 10^8^ bases) despite the lack of statistical significance. No apparent effects of DSS were observed on the other DNA adducts. Although DNA was sampled two–three days after the last DSS treatment (Fig. 1), it appears that the DNA adducts were removed by DNA repair or that damaged cells were turned over by the time of DNA sampling.

**Table 5:** DNA adducts in colon of male and female C57BL/6 mice

Experiment	DSS(%)	Days after the last DSS treatment	Male or female	Number of mice	Number of DNA adducts per 10^8^ bases			
					8-oxo-dG	εdA	8-OH-AdG	HεdC
Experiment 1–1	0%	ー	Male	4	93 ± 14 (4/4)	1.0 ± 0.1 (3/4)	1.0 ± 0.1 (4/4)	2.1 ± 0.6 (4/4)
	1cycle, 1.5%	3 days	Male	4	86 ± 23 (4/4)	1.0 ± 0.1 (3/4)	1.1 ± 0.1 (4/4)	1.3 ± 0.1 (3/4)
	1cycle, 2%	3 days	Male	4	93 ± 38 (4/4)	1.1 ± 0.4 (4/4)	1.2 ± 0.2 (4/4)	1.7 ± 0.5 (2/4)
	1cycle, 2.5%	3 days	Male	4	89 ± 21 (4/4)	1.6 ± 1.1 (3/4)	1.2 ± 0.1 (4/4)	1.3 ± 0.3 (2/4)
	0%	ー	Female	4	106 ± 5 (4/4)	0.8 ± 0.2 (2/4)	1.2 ± 0.1 (4/4)	1.9 ± 0.2 (4/4)
	1cycle, 1.5%	3 days	Female	4	87 ± 22 (4/4)	0.7 ± 0.3 (4/4)	1.1 ± 0.1 (4/4)	1.4 ± 0.5 (4/4)
	1cycle, 2%	3 days	Female	4	99 ± 22 (4/4)	1.1 ± 0.3 (3/4)	1.1 ± 0.1 (4/4)	1.4 ± 0.2 (4/4)
	1cycle, 2.5%	3 days	Female	4	81 ± 10 (4/4)	0.9 ± 0.4 (3/4)	1.2 ± 0.1 (4/4)	1.5 ± 0.5 (4/4)
Experiment 1–2	0%	ー	Male	2	126 ± 42 (2/2)	ND	1.3 ± 0.1 (2/2)	2.3 ± 0.5 (2/2)
	2cycle, 2%	2 days	Male	4	87 ± 15 (4/4)	0.8 ± 0.0 (2/4)	1.3 ± 0.3 (4/4)	1.4 ± NA (1/4)
	0%	ー	Female	2	107 ± 22 (2/2)	ND	1.1 ± 0.2 (2/2)	1.6 ± 0.2 (2/2)
	2cycle, 2.5% → 2%	2 days	Female	3	97 ± 28 (3/3)	1.4 ± 0.8 (3/3)	1.2 ± 0.2 (3/3)	2.2 ± 1.4 (3/3)

The data represent as follows: mean ± SD (number of mice in which adduct detected/number of mice tested)

Means and SDs were calculated for samples in which adduct was detected

The approximate detection limits of 8-oxo-dG, εdA, 8-OH-AdG and HεdC in the clean standards are 25 pM, 5 pM, 5 pM, 5 pM, respectively, but in real samples they increase to 30 pM, 6.3 pM, 12 pM, 8.2 pM due to ion suppression. Converting this using the average of the measured DNA amounts analyzed in this study (dG:375 μM), the detection limits of number of adducts per 10^8^ bases are 8-oxo-dG (1.7), εdA (0.37), 8-OH-AdG (0.7)and HεdC(0.48)

*Abbreviations*: 8-oxo-dG 8-oxo-7,8-dihydro-2'-deoxyguanosine, εdA, *1 N*^6^-etheno-2'-deoxyadenosine, 8-OH-AdG 8-hydroy-1, *N*^2^-propanodeoxyguanosine, HεdC heptanone-etheno-2'-deoxycytidine, ND Not detected, NA Not applicable

## Discussion

The integrity of genomic DNA is essential for life. Chronic inflammation is a major challenge for humans because inflammation induces DNA damage and effectively promotes the transformation of initiated (mutated) cells into cancerous cells [34, 35]. This study examined whether Pol κ suppresses inflammation-induced carcinogenesis by treating *Polk*^−/−^ and *Polk*^+/+^ mice with DSS. DSS treatment strongly induced colitis in both *Polk*^−/−^ and *Polk*^+/+^ mice. The severity of colitis activity indices, i.e., blood stool, soft stool and duration of decreased body weight, was higher in *Polk*^−/−^ than in *Polk*^+/+^ mice, although the differences were statistically insignificant (Table 1). These results suggest that Pol κ may play a suppressive role in inflammation. DSS induced not only colitis but also multiple dysplastic lesions and one adenocarcinoma in the colon (Table 2). The multiplicity of dysplasia was higher in *Polk*^−/−^ than in *Polk*^+/+^ mice, both in males and females, and the difference in females was statistically significant. Since β-catenin was localized in the nuclear and/or cytoplasmic regions in some dysplasias, they are considered to appear as neoplastic lesions (Figs. 3D-G). In addition, we previously reported that some dysplasias develop into adenocarcinoma through adenomas in a time-course study on colon tumorigenesis that was initiated by BP and promoted by DSS [28]. Therefore, we suggest that Pol κ suppresses inflammation-induced dysplasia, which may develop into tumors, in the colon of mice.

A possible reason for the suppressive effects of Pol κ on inflammation-induced tumorigenesis is that Pol κ suppresses inflammation-induced mutagenesis in the colon. To confirm this possibility, we analyzed mutations in the colon with the *gpt* reporter gene in female *Polk*^−/−^ and *Polk*^+/+^ mice, with or without DSS treatment. Female mice were chosen because we examined mutations in male mice in a previous study, where male *Polk*^−/−^ mice exhibited significantly higher MFs than *Polk*^+/+^ mice when they were treated with DSS (19.8 × 10^–6^ in *Polk*^−/−^ versus 6.4 × 10^–6^ in *Polk*^+/+^) [24]. MF (6.4 × 10^–6^) was not significantly different from that of vehicle-treated mice (5.6 × 10^–6^), indicating that no mutations were induced by DSS treatment in male *Polk*^+/+^ mice [24]. As shown in Table 3 and Supplementary Fig. 1, the MF in female *Polk*^−/−^ mice was more than two times higher than that in *Polk*^+/+^ mice when they were treated with DSS (10.3 × 10^–6^ in *Polk*^−/−^ versus 4.0 × 10^–6^ in *Polk*^+/+^). The MF (4.0 × 10^–6^) was not significantly different from that of vehicle-treated *Polk*^+/+^ mice (4.4 × 10^–6^). Although no statistical significance was observed, the MF of DSS-treated *Polk*^−/−^ mice (10.3 × 10^–6^) was almost two times higher than that of vehicle-treated *Polk*^−/−^ mice (5.2 × 10^–6^). Therefore, we suggested that DSS induced mutation in female *Polk*^−/−^ mice but not in *Pol*^+*/*+^ mice. Complete suppression of inflammation-induced mutagenesis may contribute to the suppression of colon dysplasia formation in *Polk*^+/+^ mice.

Mutation spectrum analysis indicated that A:T to T:A, A:T to G:C, one-base deletion, and G:C to T:A were more than three times more frequently induced by DSS in *Polk*^−/−^ than in *Polk*^+/+^ mice (Table 4). It has been reported that εdA induces A:T to T:A transversion and A:T to G:C transition [36] and 8-oxo-dG induces G:C to T:A transversions [37]. A one-base deletion was also observed in DSS-treated male *Polk*^−/−^ mice [24]. The amount of εdA in DNA was likely to increase in female C57BL/6 mice treated with DSS but insufficient to account for the mutations observed in DSS-treated *Polk*^−/−^ mice (Table 5). In addition, there is no in vitro evidence that Pol κ efficiently bypasses εdA in DNA [38, 39]. Hence, it is still an open question what DNA lesions are bypassed by Pol κ in an error-free manner, thereby suppressing DSS-induced mutagenesis.

Another possible reason for the suppressing inflammation-induced tumorigenesis in *Polk*^+/+^ mice is that Pol κ suppresses DSS-induced colon inflammation (Table 1). Accumulating evidence suggests that cytoplasmic DNA caused by DNA damage induces innate immunity, leading to the transcription of inflammatory genes [40]. The cGAS-STING pathway is a well-characterized pathway, in which cyclic GMP-AMP synthase (cGAS) binds to cytoplasmic DNA and catalyzes the formation of cyclic GMP-AMP (cGAMP), which in turn binds to the adaptor stimulator of interferon genes (STING), leading to the upregulation of inflammation-related genes [41]. In fact, it has been reported that the loss of Pol ξ, a B-family specialized Pol, induces an innate immune response involving the cGAS-STING pathway [42]. Inflammatory hyperplasia is induced by ultraviolet (UV) light in mice that express hypomorphic Rev1, which lacks the N-terminal BRCT domain [43]. Rev1 is a specialized Y-family Pol [44]. It was initially expected that less skin cancer would be induced by UV radiation because of the low-level error-prone activity of the hypomorphic Rev1. However, skin carcinogenesis was accelerated in the mice. It has been suggested that perturbed DNA replication generates cellular DNA, which induces interleukin-6, a mediator of inflammatory hyperplasia. Therefore, we envisage that DNA replication is perturbed in *Polk*^−/−^ mice when they are treated with DSS, which results in the generation of cellular DNA or micronuclei, leading to the induction of inflammatory responses and increases in colitis symptoms (Table 1). Enhanced colitis appears to contribute to dysplasia, which was more frequently observed in *Polk*^−/−^ than in *Polk*^+/+^ mice (Table 2).

In the present study, we used male and female *Polk*^−/−^ and *Polk*^+/+^ mice and treated them with DSS. Interestingly, there were sex-related differences in the levels of colitis induction and dysplasia formation (Tables 1 and 2). Male mice were more sensitive to both DSS-induced inflammation and inflammation-induced dysplasia than female mice, regardless of *Polk* genotype. In addition, MFs are higher in males than in females in *Polk*^−/−^ mice [24] (Table 3, Supplementary Fig. 1). Previous studies have also reported that DSS-induced colitis and colitis-associated colon tumorigenesis in mice are more severe in males than in females [45, 46] and one report suggested the suppressive effects of estrogens against DSS-induced colitis in mice [45]. Mutation spectra induced by DSS-induced inflammation also differed between males and females. In male mice, G:C to C:G transversion was the most significantly increased mutation when comparing the specific MFs of *Polk*^−/−^ mice with those of *Polk*^+/+^ mice [24]. In contrast, A:T to T:A was the most significantly increased mutation in female *Polk*^−/−^ mice compared to *Polk*^+/+^ mice and the specific MF of G:C to C:G was only two times higher in *Polk*^−/−^ mice than that in *Polk*^+/+^ mice (Table 4). Thus, DNA adducts (s) that cause G:C to C:G may be more abundantly generated in the colon by inflammation in males than in females. It remains to be identified which DNA lesions are responsible for the induction of G:C to C:G mutations, which are suppressed by Pol κ in the colon of mice.

## Conclusions

In this study, we suggested that Pol κ, involved in TLS, plays an important role in the suppression of colitis-induced dysplasia and mutations. Pol κ may bypass colitis-induced DNA lesions (s) in an error-free manner, thereby shutting off the induction of mutations. What DNA lesions are bypassed by Pol κ is still an open question. We envisage that the error-free bypass may also contribute to the suppression of inflammation in the colon by preventing the generation of cellular DNA or micronucleus, which leads to inflammation. There is a genetic polymorphism in the *POLK* gene in the human population [47]. Low levels of Pol κ activity may be a risk factor for inflammation, inflammation-induced mutagenesis, and carcinogenesis in humans.

## Supplementary Information

**Additional file 1: Supplementary Table 1.** Inflammation-related clinical findings and histopathology of the colon from DSS-treated Polk+/+ and Polk-/- male and female mice.Please check additional files if captured correctly.Additionl files are captured correctly.

**Additional file 2.**

## Data Availability

All data generated or analyzed during this study are included in this published article and its Supplementary Table 1.
