# Supplementary figures and images for: DNA polymerase κ suppresses inflammation and inflammation-induced mutagenesis and carcinogenic potential in the colon of mice
Source: Genes Environ. 2023 Apr 22;45:15. doi: 10.1186/s41021-023-00272-7 (PMC10122296; doi:10.1186/s41021-023-00272-7)

## Slide 1
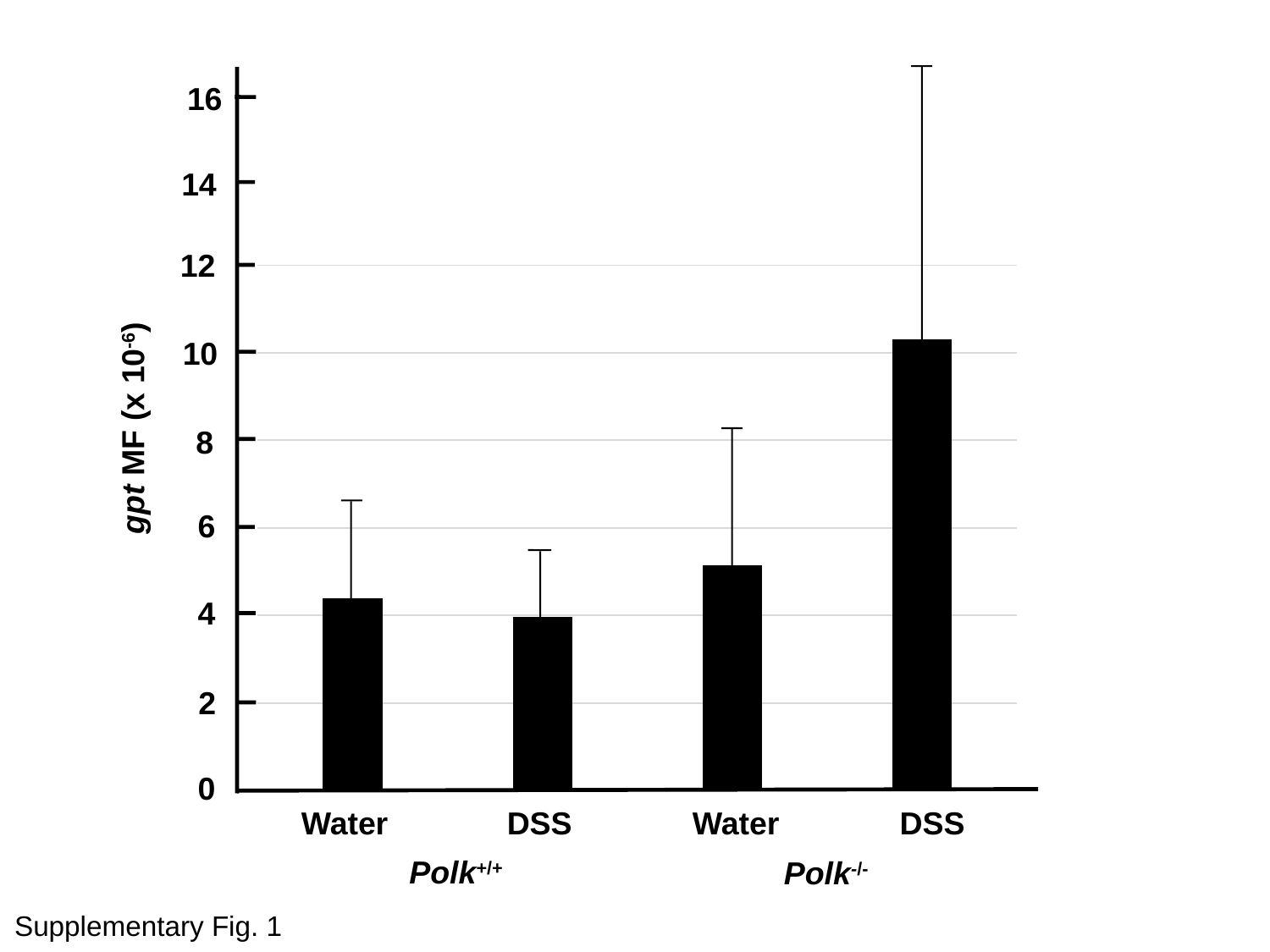

16
14
12
10
gpt MF (x 10-6)
8
6
4
2
0
Water
DSS
Water
DSS
Polk+/+
Polk-/-
Supplementary Fig. 1

Supplement: Supplementary file 2 — Additional file 2. [file 41021_2023_272_MOESM2_ESM.ppt]
